# Multiple Risk Factors of Alcoholic and Non-Alcoholic Myocardial Infarction Patients

**DOI:** 10.5539/gjhs.v8n1p62

**Published:** 2015-05-15

**Authors:** Harisharan, Awnish Kumar Singh, Nidhu Ram Dangal, Krishna Mohan Surapaneni, Ashish Joshi

**Affiliations:** 1 Saveetha Young Medical Researchers Group (SYMRG), Saveetha Medical College & Hospital, Faculty of Medicine, Saveetha University, Saveetha Nagar, Chennai, Tamil Nadu, India; 2 Operations Research in Population Health, Foundation of Healthcare Technologies Society, New Delhi, India; 3 Public Health Researcher, Department of Public Health, Foundation of Healthcare Technologies Society, New Delhi, India; 4 Department of Public Health, J.N. Medical College, KLE University, Belgaum, India; 5 Department of Biochemistry, Saveetha Medical College & Hospital, Faculty of Medicine, Saveetha University, Saveetha Nagar, Chennai, Tamil Nadu, India; 6 Department of Public Health, Foundation of Healthcare Technologies Society, New Delhi, India; 7 Department of Public Health, CUNY School of Public Health, New York, USA

**Keywords:** risk factors, myocardial infarction, alcoholics, non-alcoholics

## Abstract

**Background:**

Myocardial infarction (MI) is one of the most critical medical emergency and contributor to morbidity and mortality worldwide. Myocardial infarction is the most common form of coronary heart disease and leading cause of premature death. Past century has seen substantial advancement in the field of medical sciences but still mortality trends due to myocardial infarction is increasing in developing countries including India. We have conducted this study to compare the Sociodemographic characteristics of alcoholic and non alcoholic MI patients admitted in coronary care unit of Saveetha Medical College, Chennai, India.

**Methods:**

An exploratory cross sectional study was performed by enrolling a convenient sample of 100 Myocardial Infarction patients. Information about Sociodemographic characteristics, past medical history, alcohol and tobacco intake, physical activity, psychological stress and biochemical measurements was gathered.

**Results:**

The mean age of the respondents was 46 (SD=6) years and majority of them were male i.e. 82%. 100% married and 89% literate, there were 24% past and 22% present alcoholics. Consumption of alcohol on a monthly, weekly and daily basis was 8%, 11% and 5% respectively. Preference to brandy was 67%, rum was 21% and that the beer was 12%. Current smoker were 20% and former were 11%. 93% and 52% respondents were under medication of beta blocker and angiotensin-converting-enzyme (ACE) inhibitors respectively.

**Conclusion:**

Worldwide, MI is the most common cause of mortality and morbidity and hence early diagnosis and management is most essential. Results from our study revealed that, participants had sedentary lifestyles where risk factors of MI such as alcohol consumption, and smoking does existed.

## 1. Introduction

Myocardial infarction (MI) is caused by insufficient oxygen supply to cardiac muscles due to spasm, atherosclerosis or any kind obstruction to blood flow of coronary arteries. Individual having MI will present with retrosternal pain which may radiate to left arm. Associated symptoms involve dyspnoea, arrhythmia, perspiration, anxiety, nausea and vomiting. In some cases myocardial infarction occurs without any symptom and it is estimated to as high as 64% (Valensi, Lorgis, & Cottin, 2011). American College of Cardiology and the European Society of Cardiology published a consensus document in 2000, regarding the new definition of myocardial infarction (Antman et al., 2000). It incorporates change in level of biochemical markers of myocardial necrosis along with ischemic symptoms or electrocardiography (ECG) changes or coronary intervention (Roger, 2007). Risk of myocardial infarction is reduced among healthy population on consuming moderate amount of alcohol (Criqui, 1996; Rimm et al., 1996). Most of the studies show lowering in cardiovascular mortality among participants with previous MI, who consume moderate amount of alcohol (Mukamal et al., 2001; Janszky et al., 2008; Muntwyler et al., 1998; De Lorgeril et al., 2002; Brügger-Andersen et al., 2009). On the other hand some of the studies do not show alteration of risk among moderate drinkers. (Shaper & Wannamethee, 2000; Aguilar et al., 2004; Carter et al., 2010)

It is estimated that in 2008, 17.3 million people died due to cardiovascular diseases. Countries coming under low and middle income group are largely afflicted, where 80% of the global deaths occur. Total number of deaths from cardiovascular diseases is expected to rise to 23.3 million by 2030 (World Health Organization [WHO], World Heart Federation [WHF] and World Stroke Organization [WSO], 2011). It is most frequent cause of death around the world except in Africa (WHO, 2011). There are between 200 and 800 new cases of myocardial infarction (MI) per 100,000 men aged between 35 and 64 years annually in Europe (Helmut et al., 2007). In low and middle income countries, 29% of the deaths due to NCDs occur in individuals less than 60 years of age and in high income countries only 13% of the deaths occur under the age of 60 years (WHO, 2010, 2011). According to global burden of disease evaluation, (WHO, 2008) from the total of 751 million years living with disability (YLD) worldwide, 68% is attributable to NCDs. Ischemic heart disease is responsible to 62587 million DALYs (WHO, 2008, 2009). The south Asian countries namely India, Pakistan, Bangladesh, Sri Lanka and Nepal contribute the highest proportion of the burden of cardiovascular diseases compared with any other region of the globe as it accounts for about a quarter of the world’s population. Myocardial infarction is the most common form of coronary heart disease and leading cause of premature death (Prashant et al., 2007; Pais et al., 1996).

In India, 31.7% of mortality is attributed to MI. Incidence of cardiovascular disease has risen from 7% in 1970 up to 32% in 2011 (Reddy, 2007; Mohanasrinivasan et al., 2013). The mammoth load of CAD in India can be attributed to high exposure to risk factors like sedentary life style, smoking, alcohol, diabetes, high cholesterol level, hypertension and minimal intake of fruits in diet (Pandey et al., 2012; Goyal & Yusuf, 2006). Consumption of alcohol among in adults and even in children is increasing very rapidly in all parts of the country. This can be attributed to easy accessibility and lifestyle changes. Present study is to compare the association of risk factors of MI among alcoholic and non alcoholic MI patients. While comparing the association of risk factors opportunity was utilized to provide health education regarding the risk factors of MI and its prevention.

## 2. Methods

A cross sectional study was performed by enrolling a convenient sample of 100 Myocardial Infarction patients. Individuals of age 35 through 55 years were enrolled during inpatient stay at Intensive care unit of Saveetha Medical College, Chennai (a metropolitan city in southern state of India) from September through December 2013. Those individuals with mental and physical challenges or not willing to participate were excluded. The study was approved by the ethics committee of the Foundation of Healthcare Technologies Society, New Delhi (IRB#FHTS/014/2013) and conforms to the provisions of the Declaration of Helsinki (as revised in Tokyo 2004).

Information on the following variables was gathered.

a) **Socio-demographic characteristics**

Information was gathered about age (years), gender, educational status [no education, <High School, ≥ High School], marital status (single/married/divorce or separated/widow), annual household income, household location (rural/urban), type of family (joint, nuclear, extended), family size and occupation status (Kumar, Gupta and Kishore, 2012)

b) **Past Medical History**

Information about history of stroke, coronary heart disease and renal dysfunction was sought. Information was also gathered about current medication intake.

c) **Alcohol Intake and Tobacco Consumption**

Information about the frequency, brand and amount of alcohol consumption was gathered for past and present alcohol consumers. Information about the history of tobacco product consumption and severity of smoking was gathered.

**d) Physical Activity**

Information about the time spent in mild, moderate and vigorous physical activities in a day and number of days spent in a week in doing these activities was gathered.

**e) Medical and biochemical examination**

Measurements of blood pressure, pulse rate, pulse rhythm was done and the same time information about Exertional dyspnoea was gathered. Assessments of random blood sugar, high-density lipoprotein (HDL), low-density lipoprotein (LDL) and triglycerides were done. Echocardiography and ECG of all the participants were done bedside.

Additional Information was gathered about anxiety, anger and depression, by using five point Likert scale. Information was also sought for the type D personality [characterised by negative affectivity and social inhibition (Grande, Romppel, & Barth, 2012) and type B behaviour [characterised by relax manner, friendliness and patience (The American Heritage^®^ Medical Dictionary, 2007)] to see the proportion of participants falling in these categories.

### 2.1 Statistical Analysis

Descriptive analysis was performed using univariate statistics to report means and standard deviations for the continuous variable and frequency distributions for the categorical variables. T statistics and Analysis of variance was performed to see any possible difference in continuous variables. Chi-square analysis and Fisher’s exact test was performed to see the possible association between independent and dependent variables. All statistical analysis was performed using SPSS version16.

## 3. Results

The mean age of the respondents was 46 (SD=6) years and majority of them were male i.e. 82%. Proportion of the urban (67%) participants was higher than their rural (33%) counterparts. Hundred percent of the participants were married, living in nuclear families (62%) with an average family size of 4 (SD=1). Twenty five percent of the participants had education level of some college and one-third of the participants were working as a clerk. Majority of them were (94%) in socioeconomic category V with an average annual household income of INR 349000 (SD=55949) (Table 1).

**Table 1:** Association of the Independent variables with consumption of alcohol among the myocardial Infarction patients at Saveetha Medical College and Hospital, Chennai, India

Variable	Total (n=100)	Alcoholic (n=46)	Non-Alcoholic (n=54)	p-value
**Age (Mean ± SD)**	46±6	46±7	45±6	0.52
35-39 years	25	12 (26%)	13 (24%)	0.31
40-44 years	17	8 (17%)	9 (17%)	
45-49 years	21	6 (13%)	15 (28%)	
50-55 years	37	20 (43%)	17 (31%)	
**Location**				
Urban	67	28 (61%)	39 (72%)	0.22
Rural	33	18 (39%)	15 (28%)	
**Family Type**				
Nuclear	62	26 (56%)	36 (67%)	0.003
Joint	31	20 (44%)	11 (20%)	
Extended	7		7 (13%)	
**Family size (Mean ± SD)**	4±1	4±1	4±1	0.97
2	11	7 (15%)	4 (7%)	0.58
3	37	15 (33%)	22 (41%)	
4	38	17 (37%)	21 (39%)	
≥ 5	14	7 (15%)	7 (13%)	
**Education**				
No Schooling	11	3 (7%)	8 (15%)	0.002
< High School	27	20 (43%)	7 (13%)	
≥ High School	62	23 (50%)	39 (72%)	
**Occupation**				
Unemployed	22	5 (11%)	17 (31%)	<.0001
Clerk	31	5 (11%)	26 (48%)	
Farmer	15	7 (15%)	8 (15%)	
Industrial Labor	17	16 (35%)	1 (2%)	
Others	15	13 (28%)	2 (4%)	
**Annual Household Income (INR) (Mean ± SD)**	349000±55949	339130±61385	357407±49912	0.1
≤ 300000	50	27 (59%)	23 (43%)	0.1
> 300000	50	19 (41%)	31 (57%)	
**Physical Activity days/Week (Mean ± SD)**	2±1	3±1	2±1	<.0001
None	23		23 (43%)	<.0001
One	3		3 (5%)	
Two	16	8 (17%)	8 (15%)	
Three	35	23 (50%)	12 (22%)	
Four	23	15 (33%)	8 (15%)	
**Time given for moderate physical activity in a day (Minutes) (Mean ± SD)**	17±4	18±4	15±4	0.006
≤ 10 minutes	12	4 (9%)	8 (23%)	0.02
15 minutes	40	20 (43%)	20 (59%)	
20 minutes	20	16 (35%)	4 (12%)	
≥ 25 minutes	8	6 (13%)	2 (6%)	
**Tobacco Consumption**				
Yes	31	23 (50%)	8 (15%)	<.0001
No	69	23 (50%)	46 (85%)	
**Anxiety**				
Yes	43	17 (37%)	26 (48%)	0.31
No	57	29 (63%)	28 (52%)	
**Anger**				
High	40	18 (39%)	22 (41%)	0.87
Low	60	28 (61%)	32 (59%)	
**Depression**				
Yes	16	4 (9%)	12 (22%)	0.06
No	84	42 (91%)	42 (78%)	
**Exertion Dyspnea**				
Yes	37	22 (48%)	15 (28%)	0.03
No	63	24 (52%)	39 (72%)	
**Random Blood Sugar (Mean ± SD)**	159±21	160±23	159±20	0.86
**Diabetes Category**				
Non Diabetics	10	6 (13%)	4 (7%)	0.5
Pre-diabetics/diabetics	90	40 (87%)	50 (93%)	
**Pulse Rate (Mean ± SD)**	75±9	76±8	73±9	0.05
**Blood Pressure**				
Systolic (Mean ± SD)	148±20	146±23	149±15	0.45
Diastolic (Mean ± SD)	69±7	70 ±7	67±7	0.03
**Hypertension Stage**				
Normal	4	3 (6%)	1 (2%)	
Prehypertension	19	9 (20%)	10 (19%)	0.51
Hypertension stage I	54	22 (48%)	32 (59%)	
Hypertension stage II	23	12 (26%)	11 (20%)	
**Triglycerides (Mean ± SD)**	186±19	188±19	184±18	0.32
Normal (< 200 mg/dl)	79	36 (78%)	43 (80%)	0.72
Borderline (200-400 mg/dl)	20	10 (22%)	10 (20%)	
**HDL (Mean ± SD)**	39±5	39±5	38±5	0.61
Low (≤ 35 mg/dl)	32	16 (35%)	16 (30%)	0.58
Normal (35-60 mg/dl)	68	30 (65%)	38 (70%)	
**LDL (Mean ± SD)**	161±16	160±15	161±17	0.7
Borderline (130-159 mg/dl)	48	21 (46%)	27 (50%)	0.66
High (≥ 160 mg/dl)	52	25 (54%)	27 (50%)	
**Cardiac Ejection Fraction (Mean ± SD)**	60±14	60±15	60±14	0.83
Low	8	3 (7%)	5 (9%)	
Below Normal	35	15 (33%)	20 (37%)	0.78
Normal	56	27 (60%)	29 (54%)	

**Figure 1:**
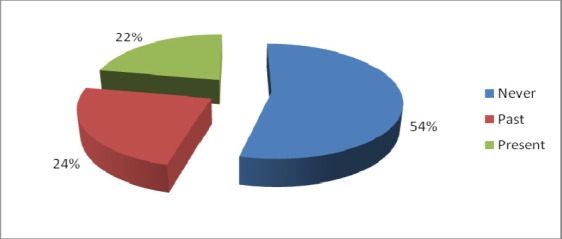
Distribution of respondents based on consumption of alcohol

**Figure 2:**
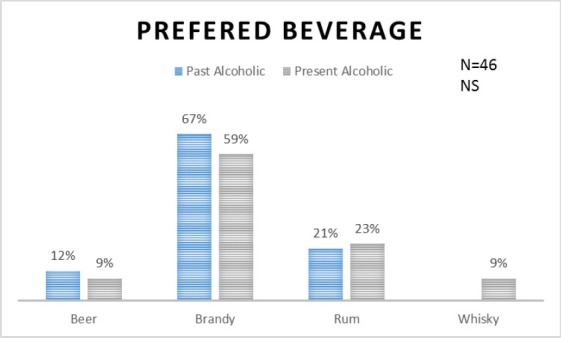
Type of alcohol beverage consumed by the past and present alcoholic patients (n=46). NS= non-significant

There were 54% respondents who never consumed alcohol, 24% were past and 22% were present consumers (Figure 1). Among 22 current alcohol consuming participants, 41% reported consumption of alcohol on weekly basis with brandy as the most preferred type of alcohol beverage. There was no significant difference in preferred type of alcohol beverage consumption in between past and present alcoholic MI patients (p=.66) (Figure 2). The average one time consumption reported by the alcohol consuming participants was 181 ml (SD=48). Sixty-nine percent of the participants had never consumed any kind of tobacco products. Among the remaining 31 participants, 68% were currently involved in one or more type of tobacco product consumption.

There were 35% respondents engaged in vigorous and moderate physical activity for 3 day in a week. The average vigorous physical activity reported by the patients was 2 days (SD=1) in a week. Likewise, all the respondents i.e. 100% reported at least 10 minutes of walking and 5 hours of sitting on weekdays.

Past medical history of the participants had shown that 2% of them had coronary heart disease (CHD) and stroke respectively and 3% had renal dysfunction in the past. Among these 7 respondents, 5% regularly adhered to the prescribed treatment plan. It was observed in the study that 48% of the participants were on angiotensin-converting-enzyme (ACE) inhibitor therapy and 93% were on beta blockers. Forty three percent of the participants had reported mild to severe anxiety and 16% reported mild depression. Six percent of the participants had type D personality and none of them had type B behaviour.

Analysis was performed to see the differences between various independent variables, including, sociodemographic characteristics, lifestyle, self reported anxiety, anger and depression, random blood sugar, blood pressure, lipid profile and cardiac ejection fraction among alcoholic and non alcoholic MI patients. There were no statistically significant differences in age, location, family size and annual household income of the alcoholic and non alcoholic MI patients. Proportion of alcoholic MI patients (56%) belonging to nuclear families was lower than Non-alcoholic MI patients (67%) and it was found statistically significant (p=.003). While There were statistically significant differences in the occupation (p<.0001), physical activity (p<.0001) and average time given to moderate physical activity in a day (p=.006). Tobacco consumers were high in proportions among alcoholic MI patients (50%) in comparison to non alcoholic MI patients (15%) which was statistically significant (p<.0001). Proportion of participants self reporting anxiety was higher among non alcoholics (48%) in comparison to alcoholic MI patients (37%) but this difference was statistically insignificant (p=.31). Self reported anger (p=.87) and depression (p=.06) had shown no significant differences among the alcoholic and non alcoholic MI patients. Exertional dyspnoea was reported to be higher among alcoholic MI patients in comparison to non alcoholic MI patients (48% vs. 28%; p=.03). There was no statistically significant difference between systolic blood pressure (p=.45) level and category (p=.51), diabetic category (p=.5), lipid profile and cardiac ejection fraction (p=.83) of alcoholic and non alcoholic MI patients (Table 1).

## 4. Discussion

Alcohol consumption is considered fifth most important risk factor for global mortality and morbidity. Consumption of alcohol and its connection with Myocardial Infarction is intricate (Leong et al., 2014). This study was performed to compare the sociodemographic characteristics, physical activity, smoking history, medication history and self reported psychological behaviour of Alcoholic and Non Alcoholic MI patients.

Previous studies had participants of average age ranging from 46 years through 65 years (Pandey et al., 2012; Abdur et al., 2005; Roy et al., 2010; Kokolis et al., 2006; Schweigman et al., 2006; Murthy et al., 2012; Abu-Tailakh et al., 2011). The average age of the participants in present study was 46 (SD=6) years which was similar to study done by Roy et al. Observations of our study had shown that 67% of the participants were from an urban setting which was dissimilar to previous study conducted by Pandey et al in which 70% of participants were from rural background (Pandey et al., 2012). Eighty two percent of the participants of the present study were male where as in previous study 30% of the participants were female (Pandey et al., 2012).

There were 22% respondents who were present alcohol consumers. Whereas previous studies reported 12.5% and 34.7% present alcohol consumers (Pandey et al., 2012; Kaur et al., 2007). Brandy (59.09%) or rum (22.72%) was the most common type of alcohol used while very low rate of whisky (9.09%) or beer consumption (9.09%) was reported in the present study among current (n=22) alcohol consumers. Likewise, study of Roy et al. (2010) revealed local/binge (56.8%) or branded spirit (52.8%) as the most common form of alcohol consumed with very low rate of wine (0.3%) or beer consumption (2.8%). There were 50.6%, and 20.2% current smokers in study carried in Libya and South India and in a case-control study showed 71.42% cases and 47.22% control current smoker (Kaur et al., 2007; Gehani et al., 2012; Rohit et al., 2012). In present study 31% of the participants were current smokers. Proportion of smokers was high among alcohol consuming MI patients and it was found statistically significant. The INTERHEART Middle East study showed greater proportion of male current and former smokers which was statistically significant (Gehani et al., 2012).

It was observed that type of family, highest education level, occupation, weekly physical activity and time given to each session of physical activity, tobacco consumption and Exertional dyspnoea had shown statistically significant differences among alcoholic and non alcoholic MI patients. Self reported anxiety and anger was higher among male in comparison to female. Blood pressure category, diabetes status category, random blood sugar, lipid profile and ejection fraction of alcoholic and non alcoholic MI patients were not statistically different.

This study had certain limitations. Firstly it was a cross sectional study with limited sample size so causality cannot be firmly established. Since it was a hospital-based study, extrapolation of the results to the wide population may not be possible. Confounding variables such as dietary pattern, genetic differences and social influences and environmental factors were not assessed.

Results from our study revealed that, participants had sedentary lifestyles and smoking also existed. Education, type of family, physical activity and occupation were significantly different among alcoholic and non alcoholic MI patients. Thus, focus need to be addressed upon screening, lifestyle interventions, and long term follow-up which will provide a chance to understand an influence of risk factors on MI outcome.

## References

[ref1] Abdur R, Mofakkarul I, Rafiqul I (2005). Selected Risk Factors for Myocardial Infarction among the Patients Admitted in Rajshahi Medical College Hospital. TAJ.

[ref2] Abu-Tailakh M, Shimon W, Yaakov H (2011). Risk Factors and Outcome of Acute Myocardial Infarction in Bedouins Living in Permanent Compared to Unrecognized Villages in Southern Israel. IMAJ.

[ref3] Aguilar D, Skali H, Moyé L. A, Lewis E. F, Gaziano J. M, Rutherford J. D (2004). Alcohol consumption and prognosis in patients with left ventricular systolic dysfunction after a myocardial infarction. J Am Coll Cardiol.

[ref4] Antman E (2000). Myocardial infarction redefined—a consensus document of the Joint European Society of Cardiology/American College of Cardiology committee for the redefinition of myocardial infarction: the Joint European Society of Cardiology/American College of Cardiology Committee. J Am Coll Cardiol.

[ref5] Brügger-Andersen T, Pönitz V, Snapinn S, Dickstein K, OPTIMAAL study group (2009). Moderate alcohol consumption is associated with reduced long-term cardiovascular risk in patients following a complicated acute myocardial infarction. Int J Cardiol.

[ref6] Carter M. D, Lee J. H, Buchanan D. M, Peterson E. D, Tang F, Reid K. J, O'Keefe J. H (2010). Comparison of outcomes among moderate alcohol drinkers before acute myocardial infarction to effect of continued versus discontinuing alcohol intake after the infarct. Am J Cardiol.

[ref7] Criqui M. H (1996). Alcohol and coronary heart disease: consistent relationship and public health implications. Clin Chim Acta.

[ref8] de Lorgeril M, Salen P, Martin J. L, Boucher F, Paillard F, de Leiris J (2002). Wine drinking and risks of cardiovascular complications after recent acute myocardial infarction. Circulation.

[ref9] Gehani A. A, Al-Hinai A. T, Zubaid M, Almahmeed W, Hasani M. R, Yusufali A. H, Yusuf S (2012). Association of risk factors with acute myocardial infarction in Middle Eastern countries: the INTERHEART Middle East study. Eur J Prev Cardiol.

[ref10] Goyal A, Yusuf S (2006). The burden of cardiovascular disease in the Indian subcontinent. Indian J Med Res.

[ref11] Grande G, Romppel M, Barth J (2012). Association between type D personality and prognosis in patients with cardiovascular diseases: A systematic review and meta-analysis. Annals of Behavioral Medicine.

[ref12] Helmut S, Angels M, Maria J. M, Montserrat C, Josep M. L, Cristina R (2007). Myocardial infarction and alcohol consumption: A population-based case-control study. Nutr Metab Cardiovasc Dis.

[ref13] Janszky I, Ljung R, Ahnve S, Hallqvist J, Bennet A. M, Mukamal K. J (2008). Alcohol and long-term prognosis after a first acute myocardial infarction: the SHEEP study. Eur Heart J.

[ref14] Kaur P, Rao T. V, Sankarasubbaiyan S, Narayan A. M, Ezhil R, Rao S. R, Gupte M. D (2007). Prevalence and Distribution of Cardiovascular Risk Factors in an Urban Industrial Population in South India: A Cross-Sectional Study. J Assoc Physicians India.

[ref15] Kokolis S, Marmur J. D, Clark L. T, Kassotis J, Kokolis R, Cavusoglu E (2006). Effects of Alcoholism on Coronary Artery Disease and Left Ventricular Dysfunction in Male Veterans. J Invasive Cardiol.

[ref16] Kumar N, Gupta N, Kishore J (2012). Kuppuswamy's socioeconomic scale: Updating income ranges for the year 2012. Indian J Public Health.

[ref17] Leong D. P, Smyth A, Teo K. K, McKee M, Rangarajan S, Pais P, INTE RHEART Investigators (2014). Patterns of alcohol consumption and myocardial infarction risk: observations from 52 countries in the INTERHEART case-control study. Circulation.

[ref18] Mohanasrinivasan V, Devi C. S, Biswas R, Paul F, Mitra M, Selvarajan E, Suganthi V (2013). Enhanced production of nattokinase from UV mutated Bacillus sp. Bangladesh Journal of Pharmacology.

[ref19] Mukamal K. J, Maclure M, Muller J. E, Sherwood J. B, Mittleman M. A (2001). Prior alcohol consumption and mortality following acute myocardial infarction. JAMA.

[ref20] Muntwyler J, Hennekens C. H, Buring J. E, Gaziano J. M (1998). Mortality and light to moderate alcohol consumption after myocardial infarction. Lancet.

[ref21] Murthy P. D, Prasad K. T, Gopal P. V, Rao K. V, Rao R. M (2012). A Survey for Prevalence of Coronary Artery Disease and its Risk Factor in an Urban Population in Andhra Pradesh. J Assoc Physicians India.

[ref22] Pais P, Pogue J, Gerstein H, Zachariah E, Savitha D, Jayprakash S, Nayak P. R, Yusuf S (1996). Risk factors for acute myocardial infarction in Indians: a case-control study. Lancet.

[ref23] Pandey S, Pandey S, Jhanwar P, Jhanwar A (2012). A prospective study of Myocardial Infarction patients admitted in a tertiary care hospital of south-eastern Rajasthan. Int J Biol Med Res.

[ref24] Prashant J, Shofiqul I, Prem P, Srinath R, Prabhakaran D, Khawar K, Yusuf S (2007). Risk Factors for Early Myocardial Infarction in South Asians Compared With Individuals in Other Countries. JAMA.

[ref25] Ram R. V, Trivedi A. V (2012). Smoking Smokeless Tobacco Consumption & Coronary Artery Disease-A Case Control Study. National Journal of Community Medicine.

[ref26] Reddy K. S (2007). India wakes up to the threat of cardiovascular diseases. J Am Coll Cardiol.

[ref27] Rimm E. B, Klatsky A, Grobbee D, Stampfer M. J (1996). Review of moderate alcohol consumption and reduced risk of coronary heart disease: is the effect due to beer, wine, or spirits?. BMJ.

[ref28] Roger V. L (2007). Epidemiology of myocardial infarction. Med Clin North Am.

[ref29] Roy A, Prabhakaran D, Jeemon P, Thankappan K. R, Mohan V, Ramakrishnan L (2010). Impact of alcohol on coronary heart disease in Indian men. Atherosclerosis.

[ref30] Schweigman K, Eichner J, Welty T. K, Zhang Y (2006). Cardiovascular Disease Risk Factor Awareness in American Indian Communities: The Strong Heart Study. Ethnicity and Disease.

[ref31] Shaper A. G, Wannamethee S. G (2000). Alcohol intake and mortality in middle aged men with diagnosed coronary heart disease. Heart.

[ref32] The American Heritage®Medical Dictionary (2007). Type B behavior.

[ref33] Valensi P, Lorgis L, Cottin Y (2011). Prevalence, incidence, predictive factors and prognosis of silent myocardial infarction: a review of the literature. Arch Cardiovasc Dis.

[ref34] World Health Organization, & World Heart Federation and World Stroke Organization (2011). Global Atlas on cardiovascular disease prevention and control.

[ref35] World Health Organization (2008). The global burden of disease: 2004 update.

[ref36] World Health Organization (2009). Global health risks: Mortality and burden of disease attributable to selected major risks.

[ref37] World Health Organization (2010). Global Status report on non-communicable diseases.

[ref38] World Health Organization (2011). Causes of deaths 2008: data sources and methods.

